# A Patient with Chronic Lymphocytic Leukemia with Pancreatic Involvement

**DOI:** 10.1155/2019/8153642

**Published:** 2019-11-16

**Authors:** Rajesh Essrani, Matthew J. Sullivan, Hiral Shah

**Affiliations:** ^1^General Internal Medicine, Lehigh Valley Health Network, Allentown, PA, USA; ^2^Department of Gastroenterology, Lehigh Valley Health Network, Allentown, PA, USA

## Abstract

A 78-year-old female with a past medical history of hypertension, type 2 diabetes mellitus, and chronic lymphocytic leukemia was hospitalized for poor appetite, weight loss, and night sweats. On physical exam, there was no palpable lymphadenopathy, and her abdomen was soft and nondistended. Laboratory results showed a hemoglobin count of 13.3 g/dl, hematocrit 41.3%, white blood cell 68.4 × 103 *μ*L with lymphocytes 92.0%, total bilirubin 0.4 mg/dL, aspartate transaminase 14 U/L, and alanine transaminase 15 U/L. CT of the chest, abdomen, and pelvis showed hypodense lesions within the pancreatic body (1.4 × 1.4 cm) and medial aspect of the pancreatic head (1.2 cm) as well as mild splenomegaly (13 cm craniocaudally). She subsequently underwent endoscopic ultrasound (EUS) with fine needle aspiration (FNA) of the pancreatic mass. Flow cytometry revealed expression of CD5 and CD23, consistent with chronic lymphocytic leukemia.

## 1. Introduction

Chronic lymphocytic leukemia (CLL) is a malignancy of CD 5-positive B cells that is characterized by the accumulation of small, mature-appearing neoplastic lymphocytes in the blood, bone marrow, and secondary lymphoid tissues. This results in lymphocytosis, infiltration of the marrow, lymphadenopathy, and splenomegaly. CLL is the most common form of adult leukemia, accounting for 10% of hematologic malignancies. CLL is diagnosed by the detection of clonal B lymphocytes at a count of ≥5,000 *μ*L in the peripheral blood, predominance of small, morphologically mature lymphocytes upon cytological examination of the blood smear, and coexpression of the B-cell antigens CD19, CD20, CD23, and T-cell antigen CD5 upon multiparametric immunophenotyping. Each clone of leukemia cells is restricted to expression of either kappa or lambda immunoglobulin light chains [1]. Due to its chronic course, patients with CLL usually do not require immediate treatment. A “watch-and-wait” strategy is the standard of care in patients with early-stage and asymptomatic CLL.

## 2. Case Report

**Figure 1:**
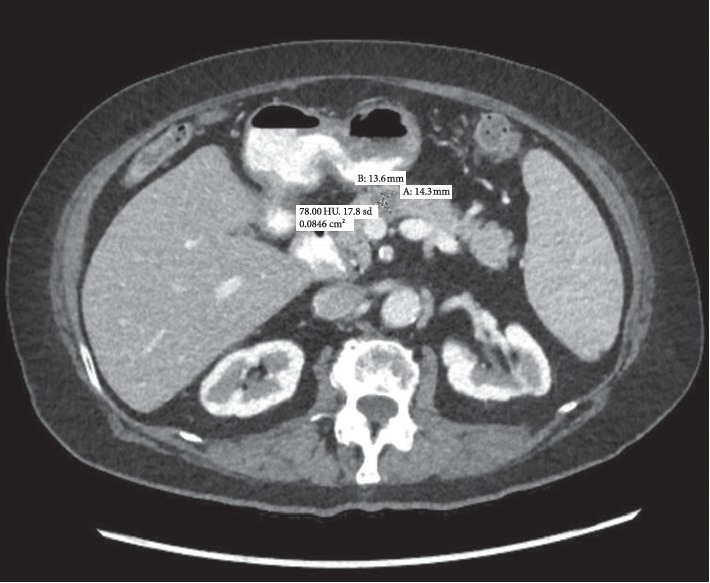
CT  abdo/pelvis: hypodense lesions within the pancreatic body and medial aspect of the pancreatic head which are concerning for a neoplastic process.

**Figure 2:**
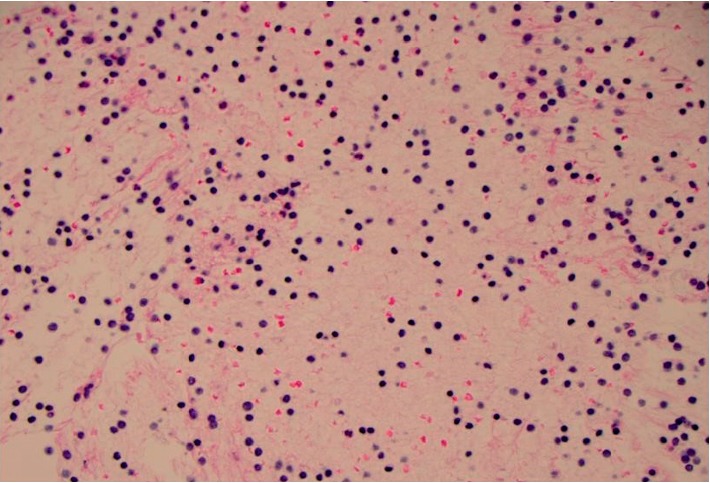
Histology slide for CLL.

A 78-year-old female with a past medical history of hypertension, type 2 diabetes mellitus, hypertension, and chronic lymphocytic leukemia was hospitalized for poor appetite, weight loss, and night sweats. On physical exam, her abdomen was soft and nondistended without palpable lymphadenopathy. Laboratory results showed a hemoglobin count of 13.3 g/dl, hematocrit 41.3%, white blood cell count 68.4 × 103 *μ*L with lymphocytes 92.0%, total bilirubin 0.4 mg/dL, aspartate transaminase 14 U/L, and alanine transaminase 15 U/L. CT of the chest, abdomen, and pelvis showed hypodense lesions within the pancreatic body (1.4 × 1.4 cm) and medial aspect of the pancreatic head (1.2 cm) as well as mild splenomegaly (13 cm craniocaudally) (Figure 1). She subsequently underwent EUS with FNA of the pancreatic mass (Figure 2). There was no blood contamination during the FNA procedure. Flow cytometry revealed coexpression of CD5 and CD23, consistent with CLL.

She was initially diagnosed with CLL around 3 years prior to the pancreatic involvement. Initial flow cytometry demonstrated a CD5- and CD23-positive clonal B-cell population. Fluorescence in situ hybridization (FISH) showed 58% of nuclear positive for apparent trisomy 12 which has been shown to correlate with an intermediate prognosis. The results for CCND1/IGH, ATM, 13q, and TP53 were all normal. Bone marrow biopsy showed 47, XX + 12 with overall pathology consistent with CLL.

**Figure 3:**
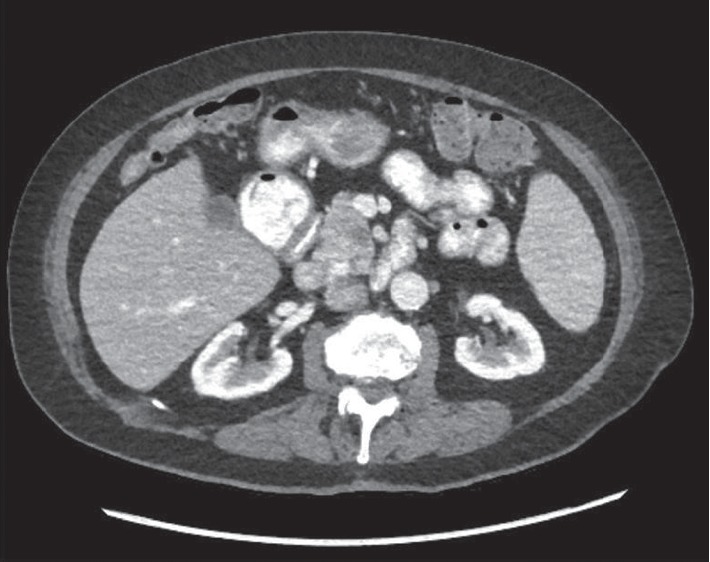
CT abdo/pelvis: normal-appearing pancreas.

After the FNA results, she was re-evaluated by her oncologist who recommended close follow-up with interval imaging in 6 months. Repeat CT scan of the abdomen and pelvis was performed two months later due to an episode of an acute abdominal pain and demonstrated a normal-appearing pancreas (Figure 3). This unexpected finding represented a spontaneous resolution of CLL involvement of the pancreas. Her suspected B-cell symptoms also resolved other than occasional fatigue reported during subsequent office visits. She has remained asymptomatic and has not required treatment of her CLL.

## 3. Discussion

Chronic lymphocytic leukemia/small lymphocytic lymphoma (CLL/SLL) is a malignancy of CD 5-positive B cells that is characterized by the accumulation of small, mature-appearing lymphocytes in the blood, bone marrow, and lymphoid tissues. The term CLL is used when the disease manifests primarily in the blood, whereas the term SLL is used when lymph nodes are mainly involved. Patients are usually asymptomatic at presentation, with CLL identified by a relative lymphocytosis on a routine complete blood count. Some patients can present with painless swelling of lymph nodes, but uncommon presentations include constitutional “B-cell symptoms” of lymphoma (i.e., fevers, chills, and weight loss), symptoms related to an acquired immunodeficiency or autoimmune complications [2].

Patients with CLL are grouped prognostically according to the Rai and Binet staging systems which are based on the physical examination and CBC results [3, 4]. The Rai system is based on the concept that, in CLL, there is a gradual and progressive increase in the body burden of leukemic lymphocytes, starting in the blood and bone marrow (lymphocytosis), progressively involving lymph nodes (lymphadenopathy), and spleen and liver (organomegaly), with eventual compromise of bone marrow function (anemia and thrombocytopenia) [5]. The Binet staging system takes into consideration five potential sites of involvement: the cervical, axillary, and inguinal lymph nodes (whether unilateral or bilateral, each area is counted as one), the spleen, and the liver. Involvement is judged only by physical exam and does not take into consideration the results of imaging studies. Patients are classified according to the number of involved sites plus the presence of anemia (hemoglobin <10 g/dL) and/or thrombocytopenia (platelets <100,000 *μ*L).

CT of the chest, abdomen, and pelvis is not routinely performed as part of the pretreatment evaluation. However, a CT scan should be done in any patient in whom enlarged abdominal or pelvic nodes are suspected based on evidence of complications such as obstructive jaundice or obstruction of the inferior vena cava or ureters [6].

Viadana et al. analyzed the involvement at every site of the human body in acute lymphoblastic, chronic lymphoblastic, acute myeloblastic, and chronic myelocytic leukemia during autopsy. CLL showed an excess of involvement in all lymph nodes (>80%), kidney (61–80%), adrenals, lungs (41–50% each), and heart (31%–40%) but rarely pancreas (11%) [7]. There was no organ involvement seen in 51–60% of autopsies. Outside of a few documented autopsy findings, there have been no case reports of CLL with pancreatic involvement reported in the literature [7, 8].

The differential diagnosis for a hypodense pancreatic lesion also includes granulomatous disease, infection, pseudocyst, cystic tumor, and solid tumors. Most pancreatic lesions are seen on CT, but MRI is more sensitive and specific.

Watchful waiting is an appropriate strategy for many asymptomatic patients with CLL. Therapy is indicated for patients with active disease as manifested by advanced stage, high tumor burden, severe disease-related “B-cell symptoms,” or repeated infections [9–12]. Early stage I and II SLL are treated with radiation therapy, and more advanced SLL is treated with chemotherapy and anti-CD 20 antibodies. Symptomatic patients should undergo a pretreatment evaluation with a complete blood count with differential and chemistries including liver and renal function and electrolytes, alkaline phosphatase, lactate dehydrogenase (LDH), beta-2 microglobulin, direct antiglobulin test (DAT), HIV, hepatitis B and hepatitis C screening, CMV serology, and fluorescence in situ hybridization (FISH) for del17p, del11q, trisomy 12, and del13q. This workup can determine the extent of disease, patient performance status, and assessment of comorbidities [2, 13, 14]. CLL patients are at increased risk of secondary malignancies like large-cell lymphoma by Richter transformation and autoimmune diseases like immune thrombocytopenia and hemolytic anemia.

In summary, CLL is usually asymptomatic on presentation and can be observed without the need for therapy for decades. There have been a few reported cases of pancreas involvement discovered at autopsy, but no case reports of symptomatic CLL involving the pancreas have been reported in the literature.
